# TAMMiCol: Tool for analysis of the morphology of microbial colonies

**DOI:** 10.1371/journal.pcbi.1006629

**Published:** 2018-12-03

**Authors:** Hayden Tronnolone, Jennifer M. Gardner, Joanna F. Sundstrom, Vladimir Jiranek, Stephen G. Oliver, Benjamin J. Binder

**Affiliations:** 1 School of Mathematical Sciences, University of Adelaide, Adelaide, Australia; 2 Department of Wine and Food Science, University of Adelaide, Adelaide, Australia; 3 Cambridge Systems Biology Centre and Department of Biochemistry, University of Cambridge, Cambridge, United Kingdom; Hebrew University of Jerusalem, ISRAEL

## Abstract

Many microbes are studied by examining colony morphology via two-dimensional top-down images. The quantification of such images typically requires each pixel to be labelled as belonging to either the colony or background, producing a binary image. While this may be achieved manually for a single colony, this process is infeasible for large datasets containing thousands of images. The software Tool for Analysis of the Morphology of Microbial Colonies (TAMMiCol) has been developed to efficiently and automatically convert colony images to binary. TAMMiCol exploits the structure of the images to choose a thresholding tolerance and produce a binary image of the colony. The images produced are shown to compare favourably with images processed manually, while TAMMiCol is shown to outperform standard segmentation methods. Multiple images may be imported together for batch processing, while the binary data may be exported as a CSV or MATLAB MAT file for quantification, or analysed using statistics built into the software. Using the in-built statistics, it is found that images produced by TAMMiCol yield values close to those computed from binary images processed manually. Analysis of a new large dataset using TAMMiCol shows that colonies of *Saccharomyces cerevisiae* reach a maximum level of filamentous growth once the concentration of ammonium sulfate is reduced to 200 μM. TAMMiCol is accessed through a graphical user interface, making it easy to use for those without specialist knowledge of image processing, statistical methods or coding.

## Author summary

Many microbes are studied by examining the colony morphology via a two-dimensional top-down image. In order to quantify such images, we typically need to label each pixel as belonging either to the colony or the background, creating a binary image. This task is laborious when performed manually and proves infeasible for large datasets. To overcome this, we have developed the software Tool for Analysis of the Morphology of Microbial Colonies (TAMMiCol), which automatically and efficiently converts colony images to binary. Multiple images may be imported and processed simultaneously, and TAMMiCol exploits the structure of the images to identify an appropriate threshold for the binary conversion of each image. The images produced by TAMMiCol, which take around 20 seconds each to process, compare favourably with images processed manually, which take anywhere up to 15 minutes, while TAMMiCol outperforms several standard image segmentation methods. After processing, the images may be exported as a CSV or MATLAB MAT file for further analysis, or may be quantified by TAMMiCol using the in-built statistics. Using TAMMiCol, we have found that colonies of *S. cerevisiae* reach a maximum level of filamentous growth once the concentration of ammonium sulfate is reduced to 200 μM.

This is a *PLOS Computational Biology* Software paper.

## Introduction

Budding yeasts, such as *Saccharomyces cerevisiae* (baker’s yeast), are able to change their pattern of growth on a solid substrate in response to the surrounding nutrient level. At sufficiently high nutrient levels, buds separate from the mother cell following cytokinesis to produce colonies that appear close to circular when viewed from above. When nutrient is not readily available, the cells reproduce via the pseudohyphal growth pattern, which is characterised by distal unipolar budding (budding opposite to the birth scar), the elongation of cells, and a persistent connection between mother and daughter cell [1]. As a result of these changes, the colony develops a number of filaments that grow along and into the substrate. This growth mode has important consequences both ecologically and medically. For example, pseudohyphal growth increases the virulence of the pathogenetic yeast *Candida albicans* [2].

Owing to the widespread occurrence of yeasts in the production of foods such as bread, wine and beer, and the need to restrict the growth of drug-resistant yeast colonies on catheters and other medical equipment [3, 4], it is important to understand and classify strain-specific properties and growth characteristics. These features are usually investigated via two-dimensional top-down images of colonies grown on a solid medium [1, 5–7]. The growth patterns observed in these experimental images are typically quantified using binary versions of the images, which indicate whether or not each pixel is part of the colony [7–9]. While it is possible to manually convert a single image to binary with sufficient accuracy using image analysis software, this task is difficult in studies of dimorphic growth, which may involve hundreds of images [7], and is infeasible for large datasets produced using genome-wide mutant libraries, which consist of thousands of images [6]. The analysis of large datasets thus requires two elements: the automated conversion of colony images to binary, and robust statistics that enable quantification of the spatial patterns.

The conversion of an image to binary requires each pixel to be placed into one of two categories (in this case, the colony and background) based upon a set of criteria. A variety of methods capable of performing this operation are available. The simplest approach to this task is thresholding, through which each pixel is categorised depending upon whether its intensity is greater than a given tolerance level. This approach is limited by the need to select a suitable tolerance, which may differ for each image and, for large datasets, must be chosen automatically to make processing feasible. There is a significant body of work regarding image thresholding and a variety of methods for achieving this are available, as illustrated in the reviews by Weszka [10], Sahoo, Soltani and Wong [11], and Sezgin and Sankur [12]. Common methods for selecting the tolerance include Otsu’s method [13], the Ridler–Calvard method [14, 15], *k*-means clustering [16, 17], a watershed transformation [18, 19] and DBSCAN [20]. Some existing techniques require particular lighting [21] or cells to be marked, such as by a fluorescent compound [22, 23], to facilitate the image analysis.

Colony identification has been performed using specialised lighting techniques combined with multilevel thresholding [24, COVASIAM], through manual thresholding on multiple layers to create three-dimensional binary images [25, COMSTAT], and by using local thresholding around areas where colonies were expected to grow [26]. Methods have also been developed to identify individual cells using a mix of different processing methods [27, 28, CellProfiler], edge detection [23, 29], Otsu’s method [21, 30] and a combination of Otsu’s method and a watershed transformation [31]. Software [32, CalMorph] and algorithms [19] tailored for yeast have also been produced for identifying and analysing individual cells. The production of binary images has been used to quantify images by examining the selected pixels in binary images produced using a range of thresholds [22]. Software for counting bacterial colonies using Otsu’s method has been developed for mobile phones [33, Colonizer].

The choice of method is dependent upon the particular application and its computational cost. The demands of processing large datasets require that the method provides no greater accuracy than is needed in order to make the analysis tractable. The statistics required thus influence the choice of method, and consideration must be given to these in order to determine the appropriate accuracy for the image processing. Previous studies of yeast colonies have relied on a variety of commercial packages to quantify the images [6]; however, these produce a limited range of statistics, such as the colony area or the change in pixel intensity pre- and post-wash. Ruusuvuori *et al*. [8] developed the web-based application Yeast Image Analysis (YIMMA) for processing and analysing images of yeast colonies, which converts images to binary representations by first applying a global intensity threshold to the green channel of a colour image, followed by post-processing to clean the image. The images are quantified by 427 features, such as the area, perimeter, and fractal dimension. Cross-validation analysis found that only 6 features were required to classify colonies as either smooth or fluffy. A set of three spatial indices designed specifically to quantify the growth of filamentous yeast colonies [7] have been shown to provide useful information on the morphology of yeast colonies and microbial mats [34, 35].

To facilitate the analysis of colony images, we have developed the software Tool for Analysis of the Morphology of Microbial Colonies (TAMMiCol). This software converts images of microbial colonies to binary for analysis using either in-build statistics or by computing other statistics after export. The binary images are produced by applying a threshold to a greyscale image. Importantly, this threshold is determined efficiently and automatically for each image by exploiting the structure of microbial colony images, which provides an advantage over generic methods for image analysis. TAMMiCol is able to process images provided there is some contrast between the colony and background, so does not require prior marking of cells and thus may be used to analyse existing datasets, as demonstrated here. While fluorescent marking is not required, any enhancements to the contrast between the colony and background may improve performance.

TAMMiCol provides several advantages over existing software used for converting images to binary, such as ImageJ [36]. Multiple images may be imported simultaneously and converted using a different threshold computed for each image without the need for the user to record macros. Post-processing steps are applied automatically and checks are performed on the binary images. TAMMiCol is able to compute specialised spatial indices [7], which are shown to be sufficiently robust so that small differences between binary images produced manually and those produced automatically do not significantly alter the statistics. Critically, use of TAMMiCol does not require knowledge of image processing and all features are accessed through a graphical user interface, placing the ability to analyse images directly with experimentalists. This package thus makes it possible to produce useful statistics automatically from experimental images alone, and opens up an avenue to study large datasets in greater detail than has previously been possible.

The images produced by this automated method are found to be of similar quality to images produced manually. Furthermore, because TAMMiCol is designed for images of microbial colonies, images produced using this method provide better agreement than images produced by standard image segmentation techniques. Through an analysis of new data using TAMMiCol, we find that colonies of the yeast *S. cerevisiae* reach a maximum level of filamentous growth once the concentration of ammonium sulfate is reduced to 200 μM. While largely applied to filamentous yeast colonies, this approach is shown to work for biofilms and other microbial colonies.

TAMMiCol is free and publicly available from github.com/HaydenTronnolone/TAMMiCol. Installation files are available for both macOS and Windows. TAMMiCol is written in MATLAB using the App Designer package and, while not requiring a MATLAB license in order to operate, does need MATLAB Runtime, which contains necessary shared libraries and will be downloaded automatically during the TAMMiCol installation. Once TAMMiCol is downloaded, installation should be complete in under five minutes. TAMMiCol does not require additional plugins and is designed for microbial colony analysis, so provides seamless operation.

## Design and implementation

### Image analysis

We consider a two-dimensional experimental image with dimensions *L*_*x*_ and *L*_*y*_ in the horizontal and vertical directions, respectively. The image is first converted to greyscale and, without loss of generality, we assume that the colony is darker than the background, as the same method may be applied when the colony is lighter by inverting the image. The colony is typically located near to the centre of the image, and so is assumed to lie within a rectangle of the same aspect ratio as the original image but 10% of the size, which ensures that some part of the colony is captured. The darkest pixel ***p*** in this central rectangle is assumed to be part of the colony and the corresponding intensity of this pixel denoted *c*_*p*_. Since ***p*** lies within the colony, pixels with intensities within some integer tolerance *τ* of *c*_*p*_ that are near to ***p*** are thus also assumed to represent part of the colony. Accordingly, for each tolerance *τ* ∈ [*τ*_*s*_, *τ*_*f*_] for some chosen minimum tolerance *τ*_*s*_ and maximum tolerance *τ*_*f*_, a binary image is produced containing all pixels with intensities in the interval [*c*_*p*_ − *τ*, *c*_*p*_ + *τ*] that form a contiguous region that contains ***p***. By requiring that the colony comprises a connected region, other artefacts in the image, such as contaminants, are automatically removed.

**Fig 1:**
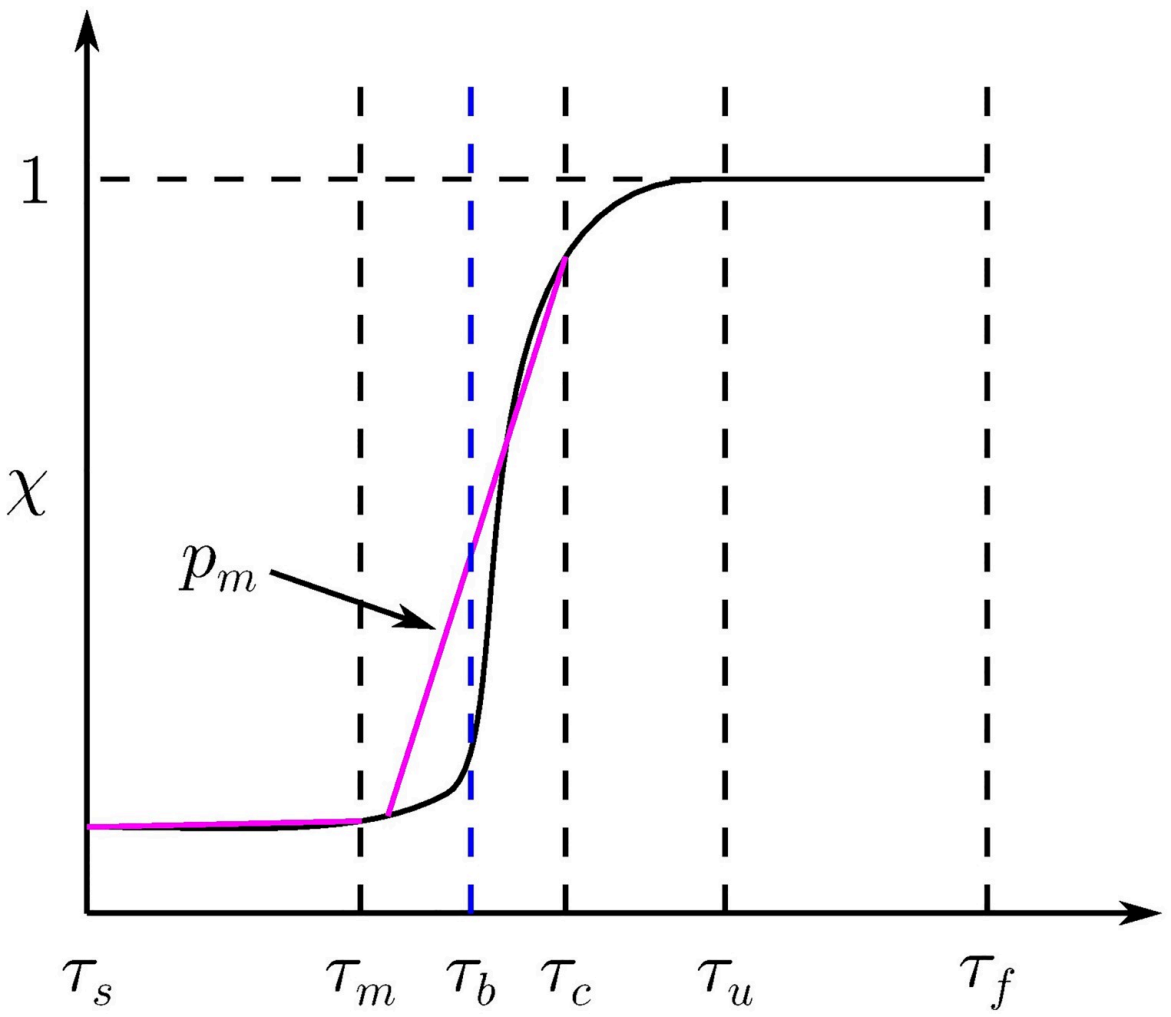
Illustration of the method used to identify the optimal tolerance *τ*_*b*_. On the interval [*τ*_*s*_, *τ*_*f*_], the proportion of selected pixels *χ* (solid black) is non-decreasing and reaches the maximum value 1 for *τ* ≥ *τ*_*u*_. The best binary image occurs at the tolerance *τ*_*b*_, which occurs just before the rapid increase in *χ*. For each *τ*_*m*_ ∈ [*τ*_*s*_ + 1, *τ*_*c*_ − 1], a piecewise linear function *p*_*m*_ (purple) is fit to *χ* on the intervals [*τ*_*s*_, *τ*_*m*_] and [*τ*_*m*_ + 1, *τ*_*c*_], for some critical value *τ*_*c*_ such that *χ*(*τ*_*c*_) is close to unity. The point *τ*_*b*_ is taken to be the threshold that minimises the mean error between *χ* and the piecewise linear interpolant.

The binary images produced for each tolerance *τ* are quantified using the proportion *χ*(*τ*) of pixels selected for each tolerance, as illustrated in Fig 1. The proportion *χ*(*τ*) is a non-decreasing function of *τ*, since increasing the tolerance can introduce additional pixels but not remove them. At low values of *τ*, the selected region will typically be a strict subset of the colony. As *τ* increases, more pixels from the colony are included in the selected region. Once *τ* becomes sufficiently large, the selected region will expand beyond the colony and start to include the background of the image. The transition as the selected region expands beyond the boundary of the colony appears as a rapid increase in the value of *χ*. Once *τ* is sufficiently large, the entire image is selected, corresponding to the maximum proportion *χ* = 1. The tolerance at which this first occurs is denoted *τ*_*u*_.

Having created a binary image for each tolerance *τ*, it remains to choose which best represents the colony. The best image is assumed to occur just before parts of the background are included at the tolerance *τ*_*b*_. This point is identified by optimising a piecewise linear fit to *χ*, as illustrated in Fig 1. A critical tolerance *τ*_*c*_ is selected from the region after the rapid increase in *χ*. In general, we choose *τ*_*c*_ = *τ*_*u*_; however, the fit may be adjusted by varying the value of *τ*_*c*_. For each midpoint tolerance *τ*_*m*_ ∈ [*τ*_*s*_ + 1, *τ*_*c*_ − 1], the proportion *χ* is approximated by a piecewise linear function *p*_*m*_(*τ*). This interpolant has two components that are defined on the intervals [*τ*_*s*_, *τ*_*m*_] and [*τ*_*m*_ + 1, *τ*_*c*_]. These are chosen to agree with *χ* exactly at the end points *τ*_*s*_ and *τ*_*c*_, and at the two adjacent values *τ*_*m*_ and *τ*_*m*_ + 1 where the intervals meet. These conditions completely specify the four coefficients of the piecewise polynomial and may be written as

$$\begin{array}{cc} p_{m}\left(\tau_{s}\right)=\chi \left(\tau_{s}\right), & \;p_{m}\left(\tau_{m}\right)=\chi \left(\tau_{m}\right),\\ \;p_{m}\left(\tau_{m}+1\right)=\chi \left(\tau_{m}+1\right), & \;p_{m}\left(\tau_{c}\right)=\chi \left(\tau_{c}\right). \end{array}$$

For each choice of midpoint *τ*_*m*_, we compute the mean error *δ*(*τ*_*m*_) between the proportions *χ* and the interpolant *p*_*m*_. The optimal threshold *τ*_*b*_ is taken to be the value of *τ*_*m*_ corresponding to the minimum of *δ*(*τ*_*m*_). Although not typically needed, the tolerance may be adjusted manually. This may be required when analysing images comprising several disconnected pieces, which bring added difficultly as the increase in *χ* near the optimal choice of *τ* may be more gradual. When processing more than one image, the tolerance selection will be repeated independently for each image, so that image may thus be processed using a different tolerance. A detailed example of this process for sample 5 of the AWRI 796 50 μM dataset produced by Binder *et al*. is given in the Supplementary Material S1 Text.

Three operations are performed on the selected image. Since it is assumed that the colony lies entirely within the image, it is expected that the filtered image will not contain any selected pixels around the boundary. If boundary pixels are selected, the tolerance is decreased automatically by one unit at a time until no pixels lie on the boundary. Next, any pixels with fewer than four neighbours in the cardinal directions are removed. While this removes a small number of pixels from the colony boundary, it ensures that any stray pixels located outside of the colony are not included in the binary image. Finally, if the user has indicated that the colony is connected, the largest connected piece in the binary image is identified and all other elements are removed from the image. The binary images are then saved as either CSV or MATLAB MAT files, which may be analysed using other software or by using the statistics incorporated into TAMMiCol, described in the next subsection.

### Quantifying the morphology

Filamentous yeast colonies may be quantified using the spatial indices introduced by Binder *et al*. [7] with some modifications, explained here briefly. To validate the binary images produced by TAMMiCol, we compare the statistics computed from the automated method with those computed from manually processed images, and examine the statistics produced from new large datasets that are infeasible to analyse manually. In the following definitions, the total number of occupied pixels is denoted *ν* and the maximum colony radius, as measured from the colony centroid, is denoted *R*. From these two quantities, the average density is calculated as *ρ* = *ν*/*πR*^2^.

To quantify the distribution in the radial direction, we count the number of pixels in *n*_*r*_ concentric annuli centred on the colony. Instead of using annuli of equal width, as per Binder *et al*. [7], the annuli are chosen to each have area *A* = *πR*^2^/*n*_*r*_. The number of pixels in the *j*th annulus is denoted *c*_*r*_(*j*). This is scaled by the number of pixels expected to lie within the annulus if the pixels were distributed uniformly, yielding the scaled counts

$$f_{r}\left(j\right)=\frac{c_{r}\left(j\right)}{\rho A}=\frac{n_{r}c_{r}\left(j\right)}{\nu }.$$

The function *f*_*r*_ has mean value 1, which is not true when using annuli of equal width. The value at which *f*_*r*_(*j*) first drops below 1 is called the complete spatial randomness (CSR) radius *R*_CSR_. Using this value, the radial distribution is characterised by the index

$$I_{r}=1-\frac{R_{\text{CSR}}}{R}\in \left[0,1\right].$$

This index measures non-uniform growth in the radial direction.

The angular distribution is quantified by computing the angular distance between each pair of pixels. Since the large number of possible pairs *ν*(*ν* − 1)/2 makes this computationally expensive, we repeatedly sample *ν*_Θ_ pixels chosen randomly from the population and average over the samples. Throughout this study, we take at most 10^3^ pixels and calculate the average of 10^3^ samples. The differences are grouped into *n*_Θ_ bins with widths *π*/*n*_Θ_ and the corresponding bin counts are denoted *c*_Θ_(*j*). These counts are scaled by the expected count for a uniform distribution to give the scaled counts

$$\begin{array}{c} f_{\Theta }=\frac{2n_{\Theta }c_{\Theta }\left(j\right)}{\nu_{\Theta }\left(\nu_{\Theta }-1\right)}, \end{array}$$

which again has mean 1. Binder *et al*. [7] introduced the index *I*_Θ_ = *f*_Θ_(1), which is a measure of local aggregation. This index is largest when all the pairs lie in the first bin, in which case *f*_Θ_(1) = *n*_Θ_. An improved index is given by scaling by this maximum value, resulting in

$$I_{\Theta }=\frac{f_{\Theta }\left(1\right)}{n_{\Theta }}\in \left[0,1\right].$$

This index measures the aggregation of cells. The maximum variance of *f*_Θ_ occurs when all the pairs lie in a single bin. In this case

$$\text{Var}\left(f_{\Theta }\right)=n_{\Theta }-1.$$

The spread of the cells is measured by the index

$$I_{\text{CSR}}=\sqrt{\frac{\text{Var}(f_{\Theta })}{n_{\Theta }-1}}\in \left[0,1\right].$$

This index measures non-uniform growth in the angular direction.

For each of the indices, larger values indicate greater variation in the morphology. For the purposes of this work, each index is calculated using 200 bins. In addition to the three indices described here, TAMMiCol produces several other values that quantify the morphology, which are described in the Supplementary Material S1 Text.

## Results and discussion

### Comparison with manually processed data

To examine the performance of the automated method, we validate the binary images produced using the filamentous yeast datasets produced by Binder *et al*. [7] and summarised in Table 1. These datasets comprise 270 raw images of filamentous yeast colonies, along with binary versions of each image that were processed manually using image processing software. The binary images produced by TAMMiCol are available online [37]. All analysis by TAMMiCol was performed using the default settings.

**Table 1:** Summary of the datasets produced by Binder *et al*. [7]. Shown are the species, nutrient level (ammonium sulfate), number of samples, number of observation times, and the mean percentage difference $\bar{d}$ between the TAMMiCol and manual images, along with the corresponding mean percentage differences ${\bar{d}}_{\text{Otsu}}$, ${\bar{d}}_{\text{kmeans}}$ and ${\bar{d}}_{\text{RC}}$ when using Otsu’s method [13], *k*-means++ clustering [16, 38] and the Ridler–Calvard method [14], respectively.

Species	Nutrient	Samples	Obs.	$\bar{d}$	${\bar{d}}_{\text{Otsu}}$	${\bar{d}}_{\text{kmeans}}$	${\bar{d}}_{\text{RC}}$
AWRI 796	50 μM	10	8	5.49	13.1	12.3	15.4
AWRI 796	500 μM	9	10	3.67	8.89	5.61	6.12
AWRI R2	50 μM	10	10	6.41	20.2	12.9	14.7

**Fig 2:**
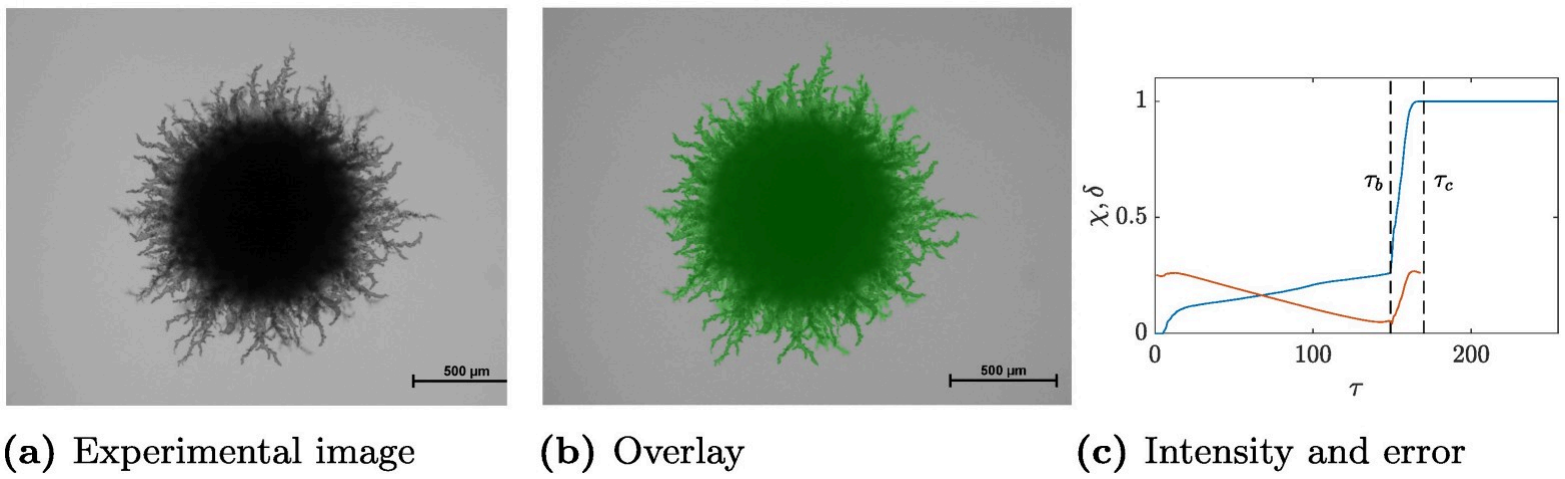
Example of image processing by TAMMiCol. (a) Colony 5 after 233 hours of growth and (b) with the selected area overlaid in green. (c) The proportions *χ* (blue) and error *δ* (red) are plotted against *τ*. Marked on this plot are the selected level *τ*_*b*_ and the critical value *τ*_*c*_. TAMMiCol is able to separate the colony from the background with a high degree of accuracy.

To first illustrate this method, we consider the experimental image of colony 5 from the AWRI 796 50 μM dataset after 233 hours of growth. The original image, the region selected by TAMMiCol and the corresponding proportions *χ* plotted against *τ* are shown in Fig 2. The selected level and the value at which all pixels are selected are also marked on this plot. The overlaid image shows that TAMMiCol is able to separate the colony from the background with a high degree of accuracy. This is confirmed by the plot of the pixel proportions, which shows that the point just before the rapid increase in the number of pixels was correctly selected.

**Fig 3:**
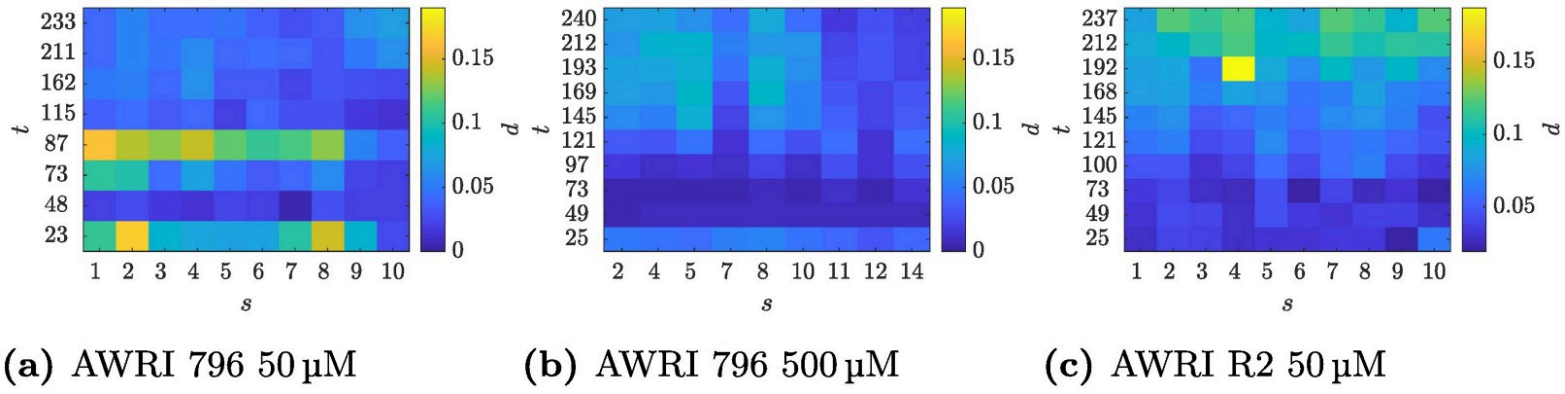
Comparison of images produced by TAMMiCol and images produced manually. The percentage difference *d* between binary images produced by TAMMiCol and binary images created manually for the (a) AWRI 796 50 μM, (b) AWRI 796 500 μM and (c) AWRI R2 50 μM datasets. The original images feature colonies of *S. cerevisiae* produced by Binder *et al*. [7] and are described in Table 1. The differences are plotted against the sample number *s* and observation time *t*. All plots are shown with the same colour scale. The difference is typically less than 10%, and many samples have disagreement values of around 5% or lower, indicating a close agreement between the automated and manual images.

The suitability of the automated method may be quantified by comparing the images produced by TAMMiCol to the images produced manually by Binder *et al*. [7]. For each pair of images, we counted both the number of pixels *ν*_*u*_ selected in the union of the manual and automated images (pixels selected as part of the colony in at least one image) and the number of pixels *ν*_*i*_ selected in the intersection (pixels selected as part of the colony in both images). A graphical representation of this process for sample 5 of the AWRI 796 50 μM dataset produced by Binder *et al*. is given in the Supplementary Material S1 Text. The relative percentage difference between the images is defined to be

$$d=\frac{\nu_{u}-\nu_{i}}{\nu_{u}}\times 100\in \left[0,100\right],$$

which is the Jaccard distance expressed as a percentage. This represents the percentage of selected pixels that differ between the two images relative to the total number of pixels that are considered part of the colony by at least one of the methods. If both images have the same set of selected pixels then *d* takes the value 0, while if there are no common selected pixels between the two images then *d* = 100. If the pixels in both images are selected at random with probability 0.5, then *d* = 200/3 ≈ 66.7. The values of *d* for each image from the datasets described in Table 1 are shown in Fig 3. For each dataset, the difference is typically less than 10% and many samples have percentage difference values around 5% or lower. Furthermore, the mean differences $\bar{d}$ over each dataset, given in Table 1, are all less than 7%. This indicates that the automated and manual images are in close agreement. Extreme value distributions [38] and Wilcoxon’s rank-sum test [39] have been used previously to perform statistical tests to determine whether agreement indicated by *d* is due to chance alone. It has been noted, however, that statistical tests performed on Cohen’s kappa coefficient, which measures agreement in a similar fashion to *d*, rarely indicate that agreement is due to chance alone [40]. As this observation also applies to *d*, we do not report formal statistical tests on these results but instead note that they indicate a high level of agreement.

To examine the performance of TAMMiCol, the trial datasets were also processed using a selection of standard methods implemented using MATLAB. The methods considered were: (1) Otsu’s method for threshold selection [13]; (2) the Ridler-Calver method [14], which is the default algorithm used by the commercial image processing software ImageJ; (3) *k*-means++ clustering [16, 41]; (4) a watershed transformation [18] using Meyer’s flooding algorithm [42]; and (5) DBSCAN [20] using natural patterns [43]. The watershed transformation (method 4) was not able to produce viable images, while DBSCAN (method 5), which has computational complexity *O*(*N*^2^) for the number of pixels *N*, proved infeasible for the image sizes considered here.

Otsu’s method (method 1), the Ridler–Calvard method (method 2) and *k*-means++ clustering (method 3) produced viable images, which were compared with the manual images in the same manner as for TAMMiCol. The respective mean differences ${\bar{d}}_{\text{Otsu}}$, ${\bar{d}}_{\text{RC}}$ and ${\bar{d}}_{\text{kmeans}}$ for these methods are given in Table 1. The mean differences for each dataset are lower for images produced by TAMMiCol than for images produced by either Otsu’s method, the Ridler–Calvard method or *k*-means++ clustering.

**Fig 4:**
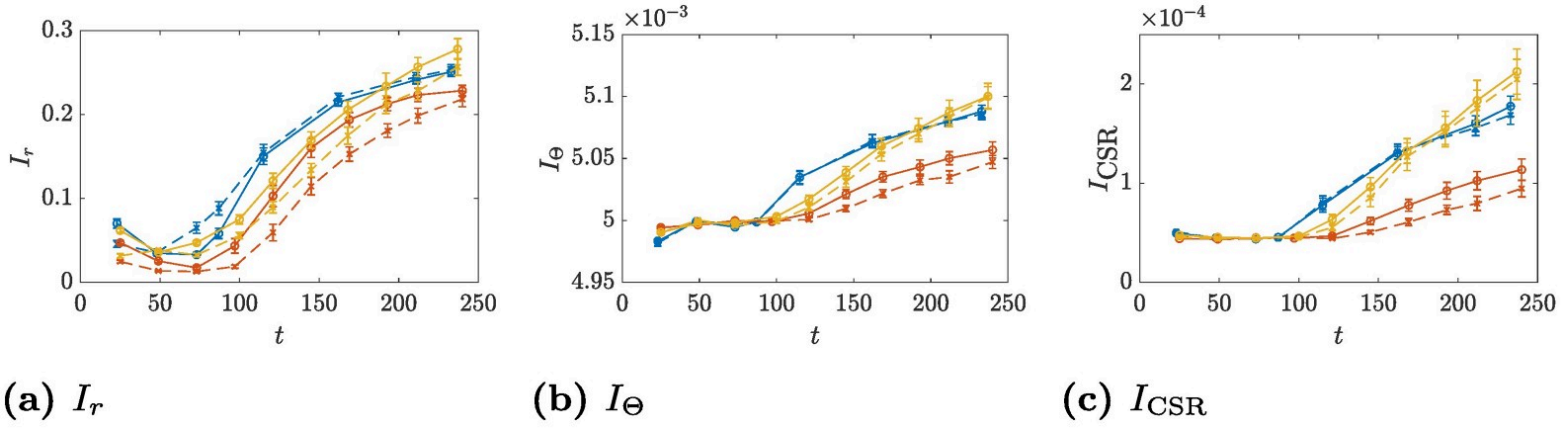
Comparison of indices computed from TAMMiCol images and manual images. The spatial indices (a) *I*_*r*_, (b) *I*_Θ_ and (c) *I*_CSR_ computed from both the TAMMiCol images (solid lines) and the manual images produced by Binder *et al*. [7] (dashed lines). Shown are AWRI 796 50 μM (blue), AWRI 796 500 μM (red), and AWRI R2 50 μM (yellow). The bars represent the standard error. The automated and manual images generally agree on the relative order of the statistics for each of the datasets.

As the images can be quantified using the in-built spatial indices, it is of interest to compare the values produced by the TAMMiCol and the manual images, which are plotted in Fig 4. Both methods produce similar results, which indicates that the automated method provides sufficiently accurate images for the computation of the indices. Importantly, despite the differences in the indices, the automated and manual images generally agree on the relative order of the statistics for each of the datasets.

### Filamentous growth and nutrient concentration

Having validated TAMMiCol using existing data, we next demonstrate the computational efficiency of this software using a larger collection of new images. It is well known that colonies of *S. cerevisiae* produce filamentous growth when starved of nitrogen [1]. In a study of 1026 strains of *S. cerevisiae*, 56% displayed filamentous growth in low-nitrogen conditions, and large variations in morphology were observed [44]. Furthermore, changes in growth have been observed due to the nitrogen source used [45]. Much work has been devoted to identifying the signalling pathways responsible for the transition to filamentous growth [46], while global gene-deletion assays have been performed to identify the genes that control filamentous growth [6]. While some studies have attempted to quantify the observed behaviour [6, 7], little quantitative information is available relating the nitrogen level and colony shape.

To address this, colonies of AWRI 796 were grown on agar at five different ammonium sulfate concentrations between 50 μM and 500 μM at 30°C, and imaged daily for ten days, as summarised in Table 2. The experiment was stopped at this point in order to avoid using images that had been distorted due to evaporation from the agar. Whereas the previous datasets considered comprised a total of 270 images and could be processed manually, the new datasets contain 690 images and, as such, manual processing of the images is infeasible. The images were thus processed using TAMMiCol only, with spatial indices computed from the resulting binary data. All analysis by TAMMiCol was performed using the default settings. Using a MacBook Pro running OS X 10.10.5 with a 2.5 GHz Intel Core i7 processor, each individual image was converted to binary and the statistics computed in approximately 20 seconds. This highlights how TAMMiCol permits analyses that were previously infeasible. The binary images produced by TAMMiCol are available online [37].

**Table 2:** Summary of new data showing the species, nutrient level (ammonium sulfate), number of samples and number of observation times.

Species	Nutrient	Samples	Obs.
AWRI 796	50 μM	14	10
AWRI 796	100 μM	14	10
AWRI 796	200 μM	14	10
AWRI 796	350 μM	14	10
AWRI 796	500 μM	13	10

**Fig 5:**
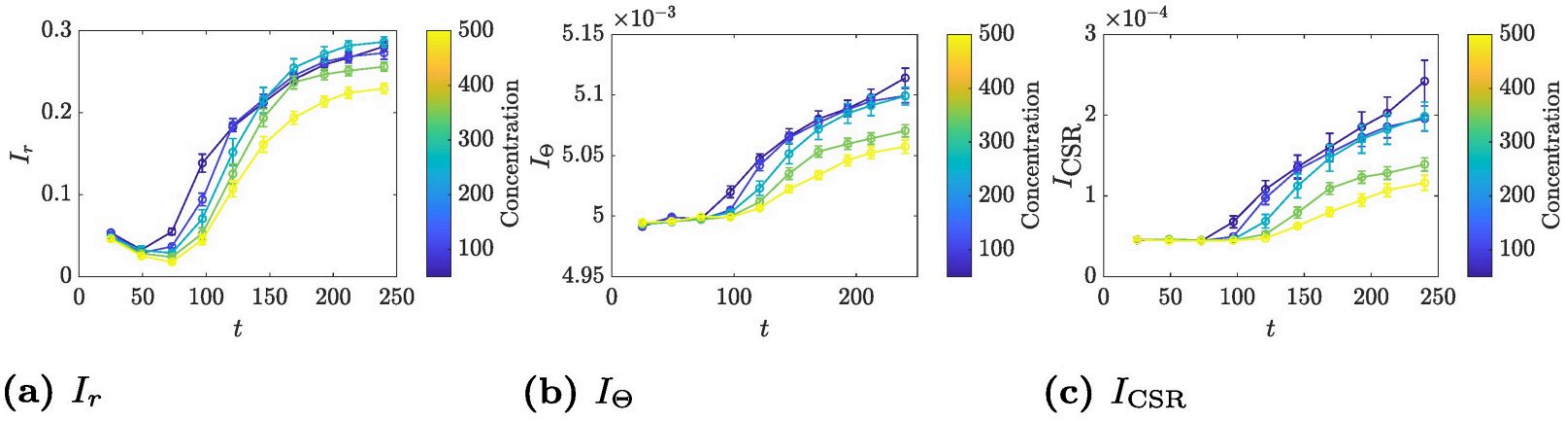
Indices for the new datasets computed using TAMMiCol. Shown are the indices (a) *I*_*r*_, (b) *I*_Θ_ and (c) *I*_CSR_ for AWRI 796 grown with ammonium sulfate concentrations of 50 μM, 100 μM, 200 μM, 350 μM and 500 μM. The bars represent the standard error. In general, the indices increase with decreasing concentration, indicating greater filamentous growth.

To compare the effect of ammonium sulfate concentration, the spatial indices *I*_*r*_, *I*_Θ_ and *I*_CSR_ were averaged over each concentration, with the resulting mean values plotted in Fig 5. In general, the indices show that filamentous growth increases with decreasing nutrient. After approximately 100 hours of growth, the colonies begin to show filamentous behaviour and the values for 350 μM and 500 μM become distinct from the other concentrations, which remain grouped closer together, suggesting that, for ammonium concentrations of 200 μM or less, the colonies reach a maximum level of filamentous growth. This indicates that a threshold concentration of ammonium is required for cells to grow in the yeast form. Below this, filamentation is triggered (presumably as a response to nitrogen stress) and some cells then switch to grow in the filamentous form. Other environmental conditions may affect the exact ammonium threshold required to trigger filamentous growth, such as the density of the medium and the concentrations of other nutrients.

### Other microbial colonies

**Fig 6:**
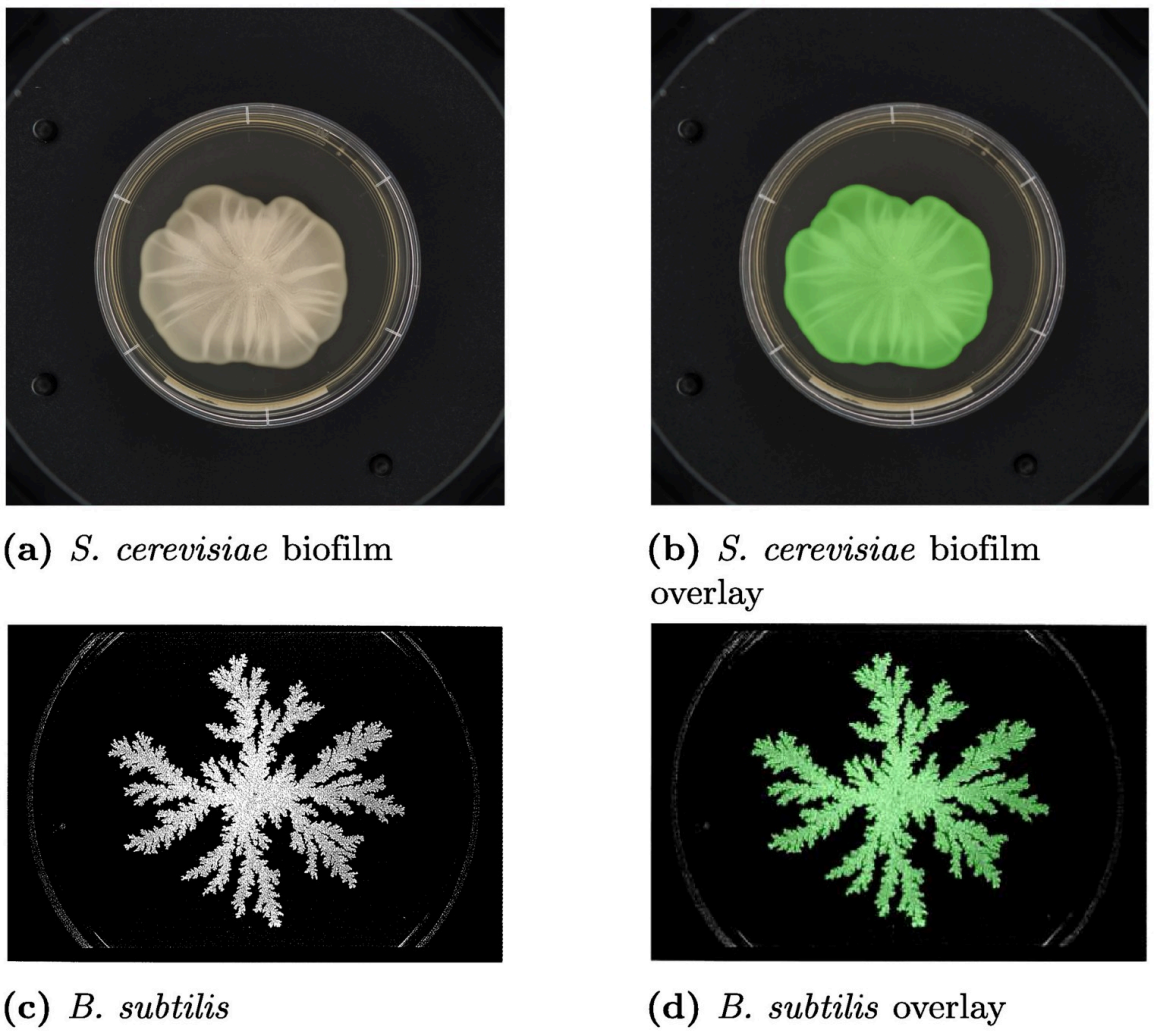
Examples of other colony types processed by TAMMiCol. Shown are (a) an *S. cerevisiae* biofilm and (c) a *B. subtilis* colony, along with the colony shown in green as identified by TAMMiCol (b and d, respectively). In each case, the colony is identified with a high degree of accuracy, demonstrating the versatility of the software. Fig. 6(a and b) was produced by Tam *et al*. [34]. Fig. 6(c and d) is reprinted from the *Journal of the Physical Society of Japan*, 58(11), Hiroshi Fujikawa and Mitsugu Matsushita, Fractal Growth of *Bacillus subtilis on Agar Plates*, 3875–3879, 1989 and is reproduced under a Creative Commons Attribution licence (CC BY 4.0).

While all the examples thus far have considered filamentous yeast colonies, the methods presented here are applicable to a variety of other cases. To illustrate this, we consider an *S. cerevisiae* (L2056) biofilm produced by Tam *et al*. [34], and a colony of *Bacillus subtilis* produced by Fujikawa and Matsushita [47], both of which are shown with the colony identified by TAMMiCol in Fig 6. In both cases, TAMMiCol is able to identify the colony with a high degree of accuracy, demonstrating the versatility of the software. The statistics used here are also suitable for analysing these colonies [34, 35].

## Availability and future directions

We have introduced the software TAMMiCol, which converts photographs of microbial colonies to binary images automatically and in a computationally efficient manner. The binary images are produced using thresholding with the tolerance chosen by exploiting the structure of the images. By tailoring the method for images of microbial colonies, TAMMiCol produces results comparable with manual image processing and better than those produced by standard image segmentation methods, while the graphical user interface gives experimentalists direct access to quantification methods without the need for specialist knowledge of image processing or coding. TAMMiCol is free and publicly available from github.com/HaydenTronnolone/TAMMiCol.

Using this method, it is possible to analyse large datasets that would be infeasible to process manually, as each image may be converted to binary and quantified in approximately 20 seconds using a laptop computer. In comparison, it can take up to 15 minutes to manually process an image. While TAMMiCol has been demonstrated using examples containing up to 690 images, the same procedure makes it possible to quantify the morphology from datasets containing thousands of images, such as genome-wide deletion mutant libraries. Therefore, TAMMiCol provides the opportunity for future research on identifying specific genes responsible for measurable growth characteristics. Through an analysis of a new large dataset using TAMMiCol, we have shown that colonies of the yeast *S. cerevisiae* reach a maximum level of filamentous growth once the concentration of ammonium sulfate is reduced to 200 μM.

TAMMiCol presents several advantages over existing software used for producing binary images, such as ImageJ [36]. The default method for producing binary images in ImageJ is through the IsoData algorithm, which is a version of the Ridler–Calvard method [14] tested here and which was outperformed overall by TAMMiCol. TAMMiCol automatically undertakes a number of steps in addition to thresholding to remove artefacts from the images. While similar steps could be performed using other software, this would need to be done manually by the user, which could be difficult without experience in image processing. TAMMiCol is designed for batch processing, while ImageJ requires the user to record a macro in order to process multiple images, and other software may lack this capability altogether. Finally, TAMMiCol is able to organise the binary output and produce statistics to describe the data. This means that TAMMiCol takes raw images as input and produces appropriate statistics in one action, so that data are available to the user without the need for specialist skills in image processing, data management or shape quantification. We believe that no other software provides the ease of use and combination of features available through TAMMiCol.

While we have shown that the indices developed by Binder *et al*. [7] are able to quantify the spatial pattern, the binary images produced by this method are not limited to these measures. The processed images may be accessed by other image-analysis software and other features examined, such as the perimeter or number of connected pieces. Furthermore, the methods described here may be used to analyse a wide variety of microbial colonies and other images with similar features. This work thus opens an avenue to efficiently quantify numerous large datasets using either the indices provided or any other custom statistics. This has the potential to provide new insights that were previously unobtainable, and to motivate new experimental work that was previously unsupported by statistical analysis.

While the current version of TAMMiCol has been designed to convert images of microbial colonies to binary, the methods employed here could be used to analyse a variety of images. This includes, but is not limited to, images of scratch assays, tumour spheroids and vegetation patterns. While TAMMiCol is expected to be able to convert other images, the method could be improved by employing alternative methods for identifying the best threshold that would provide additional user control, so that the best method could be selected. Future versions of TAMMiCol may be further generalised by incorporating a variety of algorithms to assist in selecting the best binary image, and by analysing individual colour channels rather than a greyscale image. While the indices computed by TAMMiCol have been designed to quantify microbial colonies, future versions may include additional indices and pair-correlation functions that would permit general-purpose analysis.

## Supporting information

S1 Text Supplementary material. An illustrative example showing the processing of an image using TAMMiCol, and further analysis of the statistics used.
(PDF)

S1 Code TAMMiCol source code. Source code for TAMMiCol written in MATLAB.
(ZIP)

S1 Data Test data. Sample images from a single colony for processing by TAMMiCol.
(ZIP)
