# Supplementary material for: TAMMiCol: Tool for analysis of the morphology of microbial colonies
Source: PLoS Comput Biol. 2018 Dec 3;14(12):e1006629. doi: 10.1371/journal.pcbi.1006629 (PMC6292648; doi:10.1371/journal.pcbi.1006629)
Supplement: S1 Text — An illustrative example showing the processing of an image using TAMMiCol, and further analysis of the statistics used. (PDF) [file pcbi.1006629.s001.pdf]

TAMMiCol: Tool for Analysis of the Morphology of Microbial Colonies (Supplementary Material)

Hayden Tronnolone*[1], Jennifer M. Gardner[2], Joanna F. Sundstrom[2], Vladimir Jiranek[2], Stephen G. Oliver[3], and Benjamin J. Binder[1]

[1]School of Mathematical Sciences, University of Adelaide, Adelaide, SA 5005, Australia
[2]Department of Wine and Food Science, University of Adelaide, Adelaide, SA 5005, Australia
[3]Cambridge Systems Biology Centre and Department of Biochemistry, University of Cambridge, Cambridge CB2 1GA, United Kingdom

September 3, 2018

1 Illustrative Example

The binary conversion performed by TAMMiCol is illustrated by considering the analysis of a single image in detail. We consider the image of colony 5 of strain AWRI 796 at concentration 50 µM after 233 hours of growth, produced by Binder, Sundstrom, Gardner, Jiranek and Oliver (2015). The binary conversion is illustrated in figure 1. The darkest pixel in the centre of the image is selected, which has intensity c_p. All connected pixels with intensities in the range $[c_p - \tau, c_p + \tau]$ are also selected for a range of different thresholds τ and the proportion χ of pixels selected in each case is recorded. Selected examples of the binary images produced are shown in figure 1. At low values of τ, almost no pixels are selected. As τ increases, more of the colony is selected until τ is so large that the entire image is selected, corresponding to $\chi(\tau_u) = 1$. The best value of the tolerance τ_b occurs just before the sudden jump in χ, which occurs when the background is included in the selected region. This point is identified by fitting a piecewise linear function to χ between $\tau = 0$ and $\tau_c = \tau_u$. For each choice of the discontinuity, the error δ between the fitted function and χ is computed. The value of the discontinuity that minimises the error is taken to be the optimal tolerance.

2 Additional Methods for Quantification

2.1 Angular Index

The angular distribution is further quantified by computing the angle between each selected pixel and the positive x-axis, measured with respect to the colony centroid, and grouping this data into n_θ bins each having width $2\pi/n_\theta$. The counts $c_\theta(j)$ in each bin are scaled by the expected number for a uniform distribution of pixels, yielding the normalised counts

$$f_\theta(j) = \frac{n_\theta c_\theta(j)}{\nu},$$

*Corresponding author: hayden.tronnolone@adelaide.edu.au

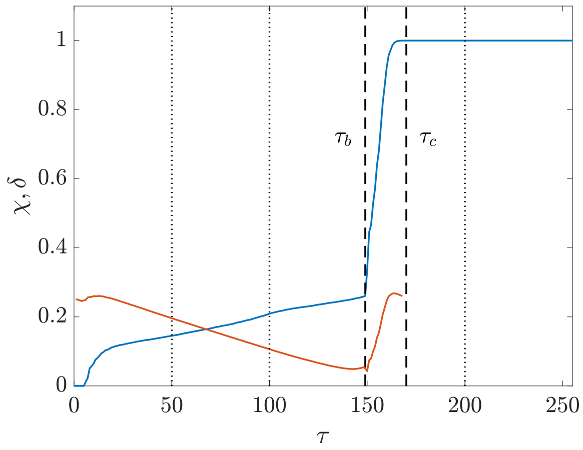

(a) Proportion χ and error δ

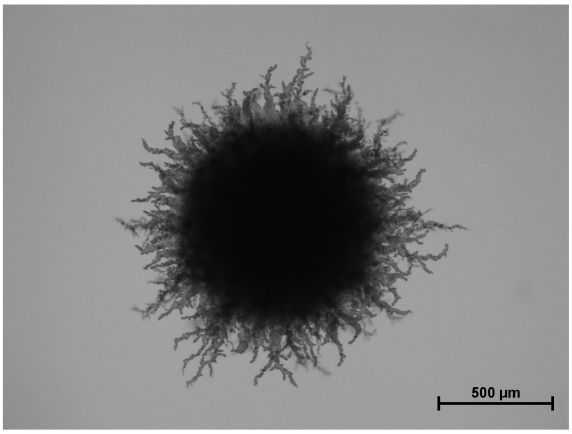

(b) $\tau = 0$

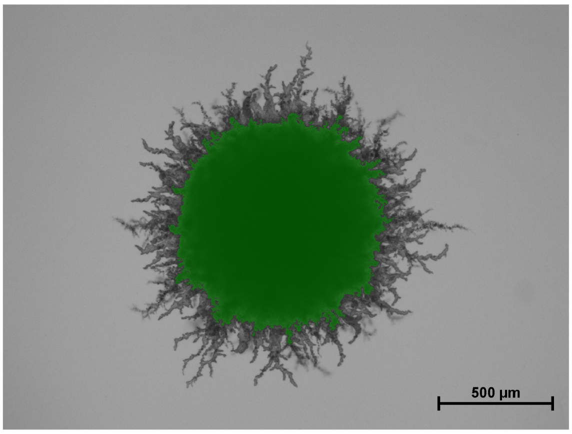

(c) $\tau = 50$

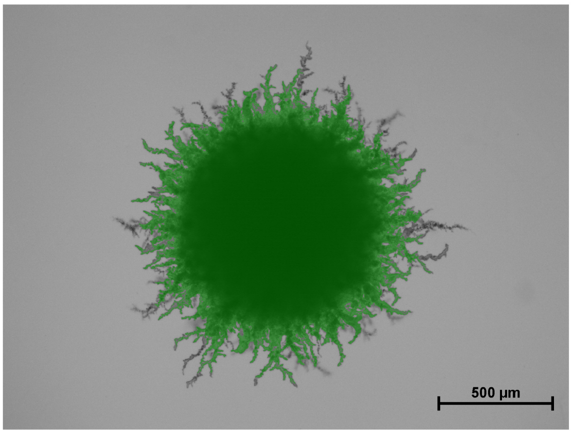

(d) $\tau = 100$

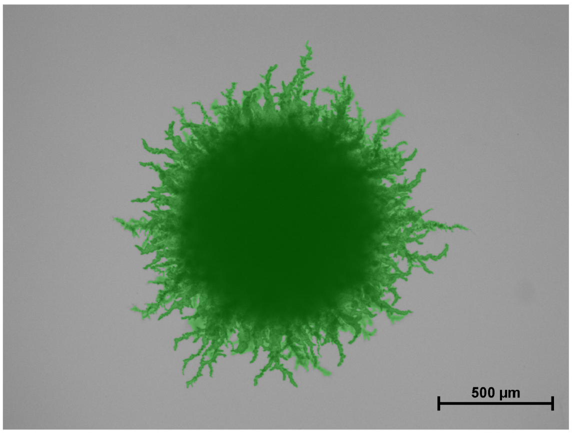

(e) $\tau = 149$

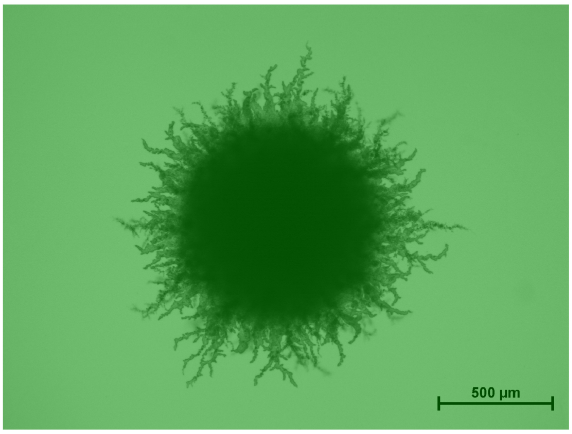

(f) $\tau = 200$

Figure 1: An illustrative example of the conversion to binary by TAMMiCol. Shown is the conversion of colony 5 after 233 hours of growth produced by Binder *et al.* (2015). (a) The proportion *chi* (blue) and error δ (red) are plotted against τ. Marked (dashed) are the best value $\tau_b = 149$, which minimises the error δ, and the value at which χ first reaches unity $\tau_u = \tau_c = 170$. The values $\tau = 50, 100$ and 200 (dotted) are also shown. The selected colony is shown at (b) $\tau = 0$, (c) 50, (d) 100, (e) $\tau_b = 149$ and (f) 200.

which again has mean 1. The function f_θ may be characterised by the standard deviation $\sigma_\theta = \sqrt{\mathrm{Var}(f_\theta)}$, and it is known that

$$\mathrm{Var}(f_\theta) = E[f_\theta^2] - E[f_\theta]^2 = \frac{1}{n_\theta}\sum_{j=1}^{n_\theta}\left(\frac{c_\theta(j)}{\nu/n_\theta}\right)^2 - 1.$$

The maximum variance occurs when all the points lie in a single bin and is given by

$$\mathrm{Var}(f_\theta) = n_\theta - 1.$$

The angular distribution is then characterised by the index

$$I_\theta = \sqrt{\frac{\mathrm{Var}(f_\theta)}{n_\theta - 1}} \in [0, 1].$$

2.2 Other Measures

In addition to the CSR radius R_{CSR}, TAMMiCol can compute an alternative minimum radius by finding the radius of the largest disk that fits within the colony. Whichever radius is chosen is denoted $R_{\min}$. The minimum radius $R_{\min}$, maximum colony radius $R_{\max}$ and colony area A are all provided by TAMMiCol.

TAMMiCol provides further description of the pair correlation function f_Θ using the Fourier transform. The coefficients of the Fourier transform are provided, along with the a list of wave numbers ordered by the magnitude of the corresponding Fourier coefficients. An example of this analysis is given in subsection 3.2.

2.3 Choice of Radius

For selected pixels $\boldsymbol{x}$, the disk $|\boldsymbol{x}| < R_{\mathrm{CSR}}$ is dominated by the solid interior of the colony, while the annulus $R_{\mathrm{CSR}} \le |\boldsymbol{x}| \le R$ is dominated by filamentous growth. Due to this, Binder *et al.* (2015) computed the angular indices using only pixels in the annulus $R_{\mathrm{CSR}} \le |\boldsymbol{x}| \le R$; however, this choice means that the indices computed may be very sensitive to the binary conversion of the image. To explore the effect of this choice, we consider annuli $\beta R_{\mathrm{CSR}} \le |\boldsymbol{x}| \le R$, where $\beta \in [0, 1]$. To separate this from the effects of the automatic image processing, we perform this analysis on manually processed images for the AWRI 796 dataset produced by Binder *et al.* (2015).

The computed indices are shown in Figure 2 for $\beta = 0, 0.1, \ldots, 1$. For $\beta < 1$, each index shows a similar behaviour qualitative relative for the different data sets. When $\beta = 1$, the indices all show a significant change. Based on this, we take $\beta = 0$ from this point onwards, which is equivalent to analysing the entire dataset, rather than considering an annular region only.

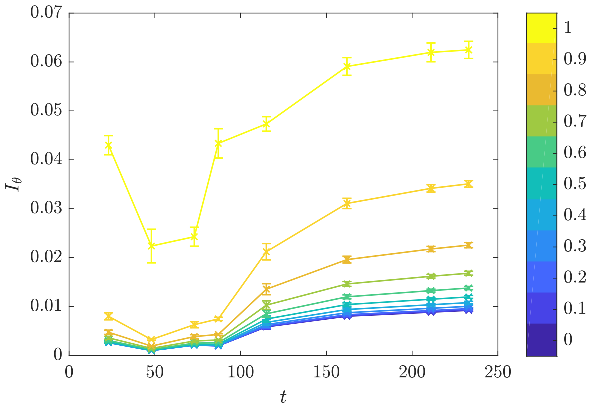

(a) I_θ

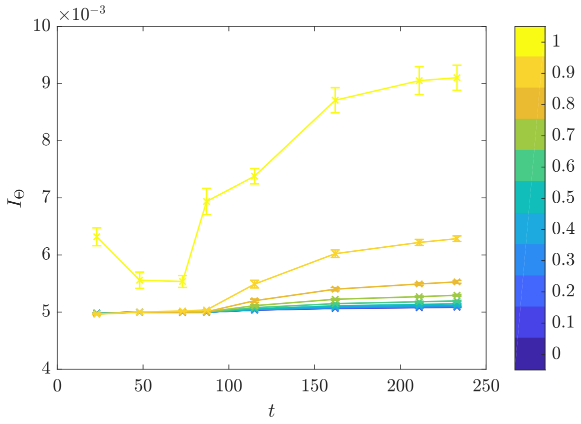

(b) I_Θ

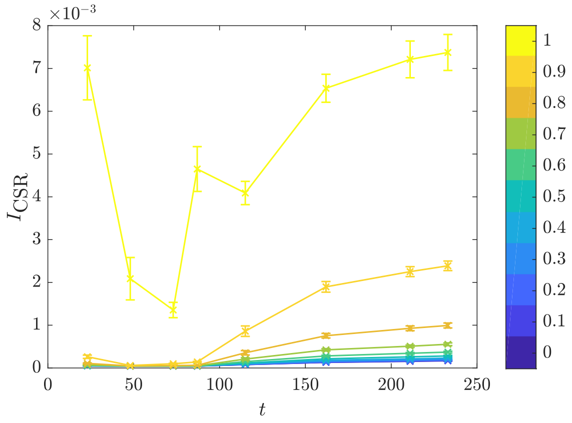

(c) I_{CSR}

Figure 2: Indices for the AWRI 796 50 µM dataset produced by Binder *et al.* (2015) computed using the manually processed images. The indices were computed using $\beta = 0, 0.1, \ldots, 1$. Although the quantitative values change with β, the qualitative behaviour is similar for $\beta < 1$. For $\beta = 1$, there is a significant relative change between the indices.

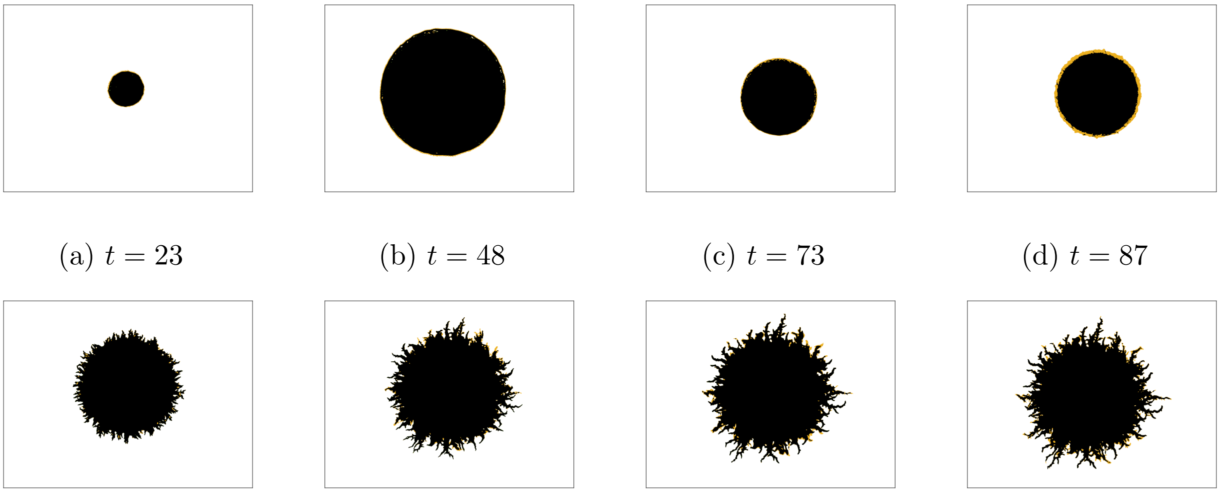

(a) $t = 23$ (b) $t = 48$ (c) $t = 73$ (d) $t = 87$

(e) $t = 115$ (f) $t = 162$ (g) $t = 211$ (h) $t = 233$

Figure 3: Comparison between the exact and processed images for colony 5 from the AWRI 796 50 µM dataset (Binder *et al.*, 2015). Times $t = 23$ and 48 are shown at a larger magnification. Pixels found in both images are coloured black, pixels only in the automated images are yellow, while pixels only in the exact images are green. The two sets of images, particularly those showing filamentous behaviour, show very good agreement, with the largest discrepancy at $t = 87$.

3 Comparisons

3.1 Comparison Images

To further evaluate the automated method we compare the results with the images of colony 5 from the AWRI 796 50 µM dataset over all time observations. The manual and automated images are overlaid in Figure 3, with the differences between each method highlighted. These show that both methods produce graphically similar results. The largest discrepancies occur at $t = 87$, although this is before any filamentous behaviour is evident. There is very good agreement at all later times.

3.2 Fourier Analysis

The two image sets may be further compared by considering the Fourier transforms F_Θ of the pair correlation functions f_Θ. To illustrate this, we consider colony 5 from the AWRI 796 strain after 233 hours of growth. The pair correlation function f_Θ and the spectra computed from the Fourier transform F_Θ are plotted in Figure 4. The averaged spectrum contains additional dominant modes when compared to the spectrum for a single colony.

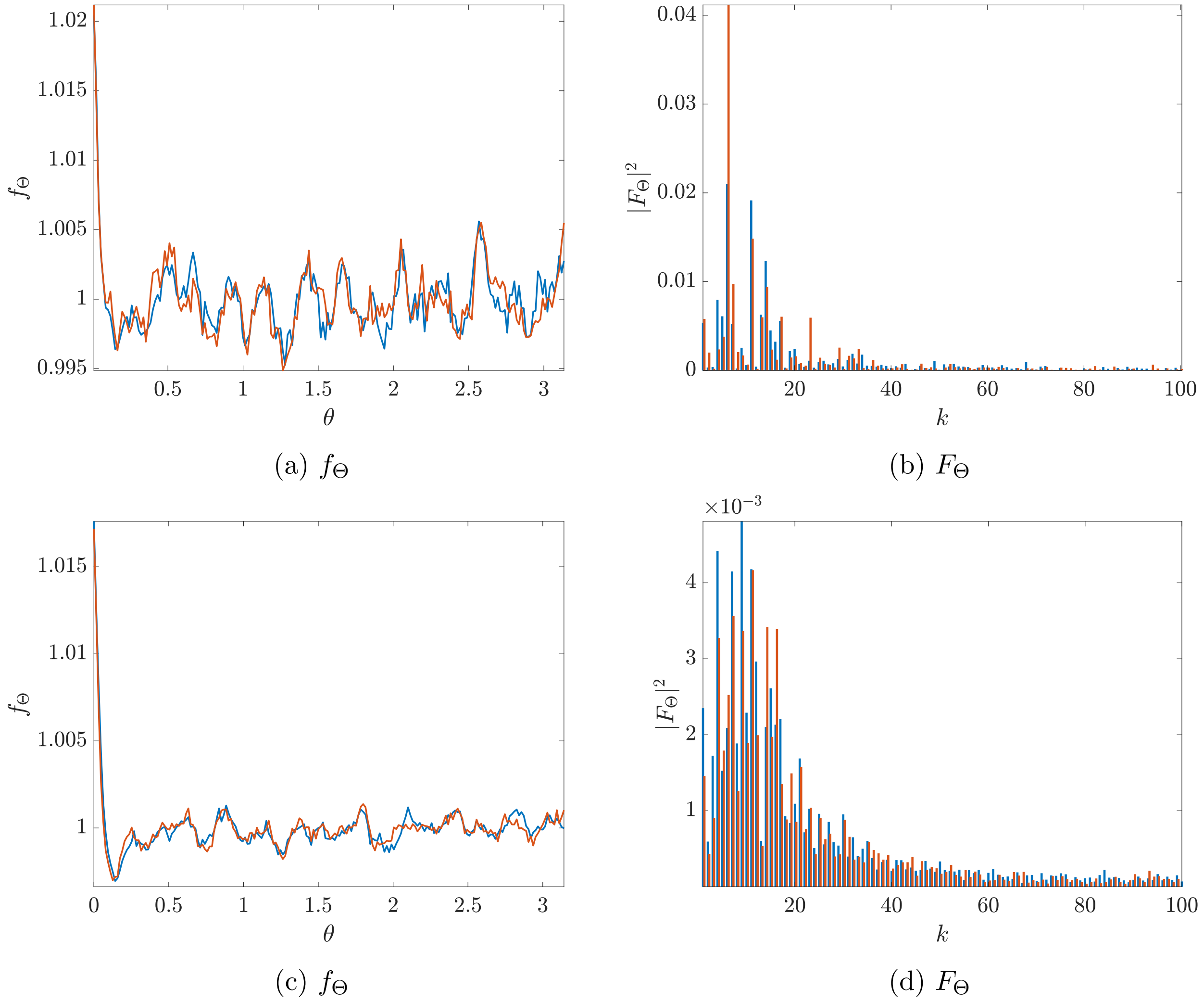

(a) f_Θ (b) F_Θ

(c) f_Θ (d) F_Θ

Figure 4: Comparison between the automated (red) and manual (blue) images for the AWRI 796 strain after 233 hours of growth. Shown are (a) the pair correlation functions f_Θ for colony 5, (b) the spectra computed from the Fourier transforms F_Θ for colony 5, (c) the average pair correlation function over all samples and (d) the average spectra.

References

Binder, B. J., Sundstrom, J. F., Gardner, J. M., Jiranek, V. and Oliver, S. G. (2015), ‘Quantifying two-dimensional filamentous and invasive growth spatial patterns in yeast colonies’, *PLOS Computational Biology* **11**(2), e1004070.
