# Supplementary figures and images for: TAMMiCol: Tool for analysis of the morphology of microbial colonies
Source: PLoS Comput Biol. 2018 Dec 3;14(12):e1006629. doi: 10.1371/journal.pcbi.1006629 (PMC6292648; doi:10.1371/journal.pcbi.1006629)

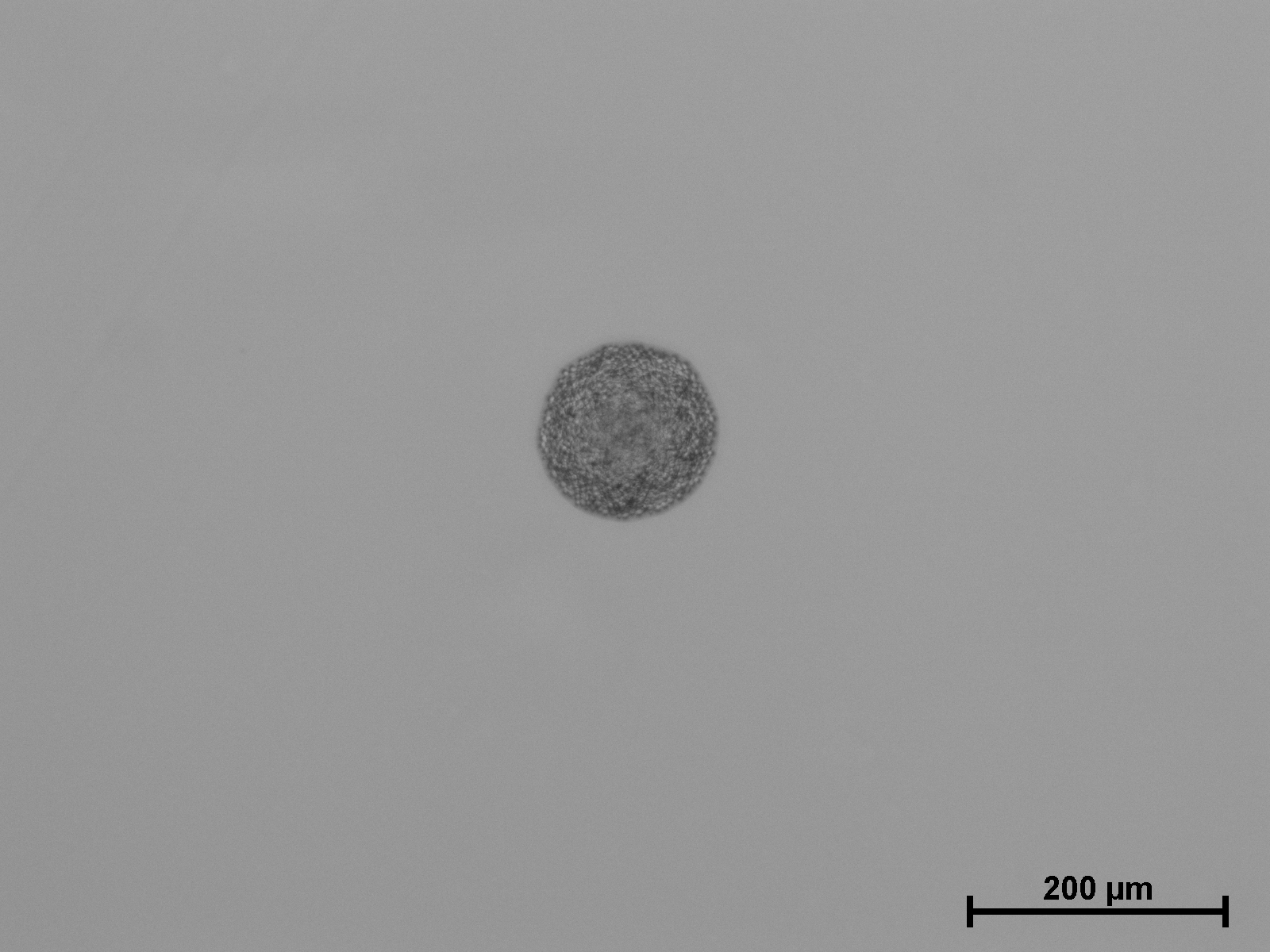

Supplement: S1 Data — Sample images from a single colony for processing by TAMMiCol. (ZIP) [file pcbi.1006629.s003.zip › Test Data/AWRI 796 PLOS 50uM s5 10X 23h.tif]

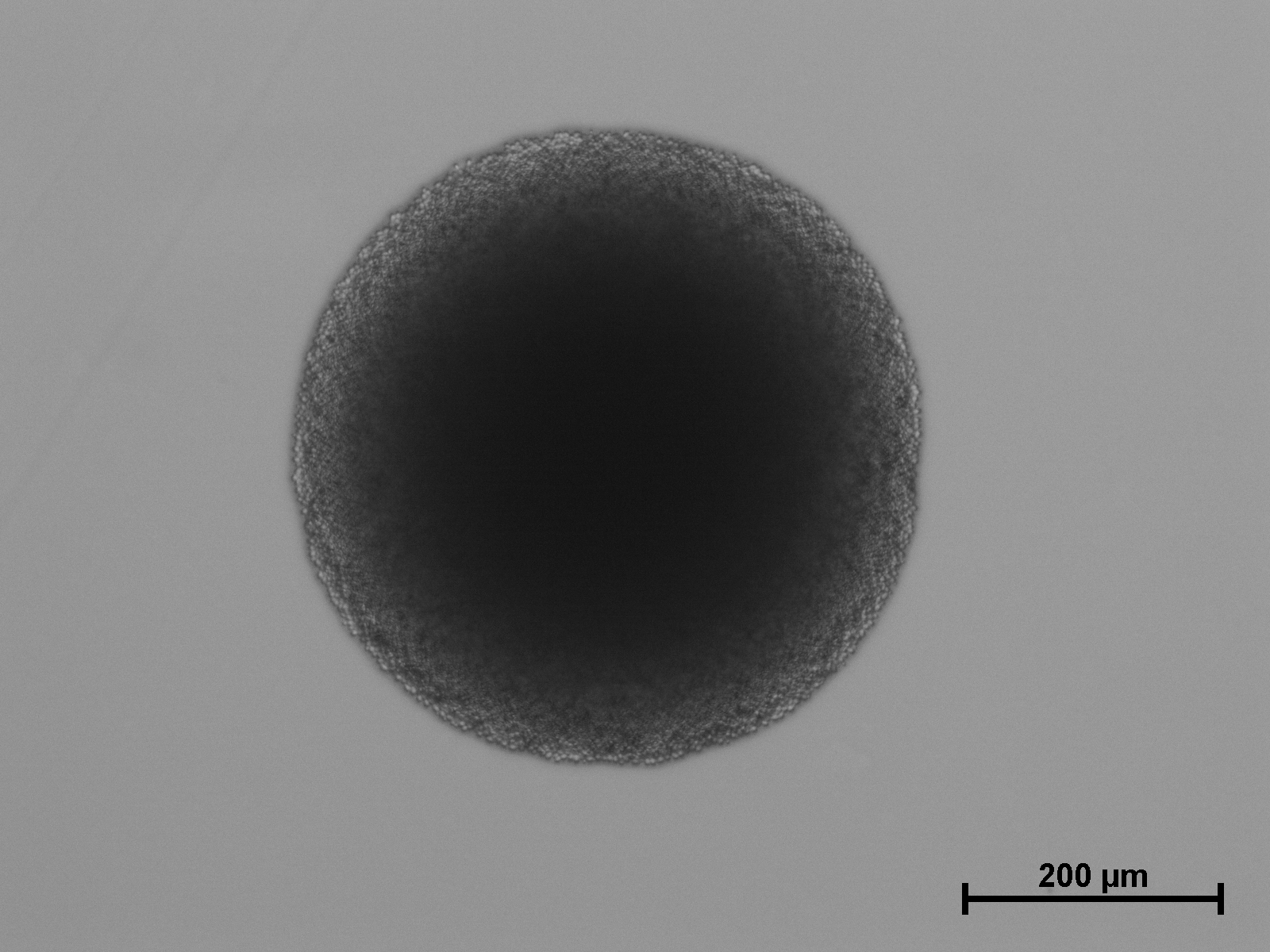

Supplement: S1 Data — Sample images from a single colony for processing by TAMMiCol. (ZIP) [file pcbi.1006629.s003.zip › Test Data/AWRI 796 PLOS 50uM s5 10X 48h.tif]

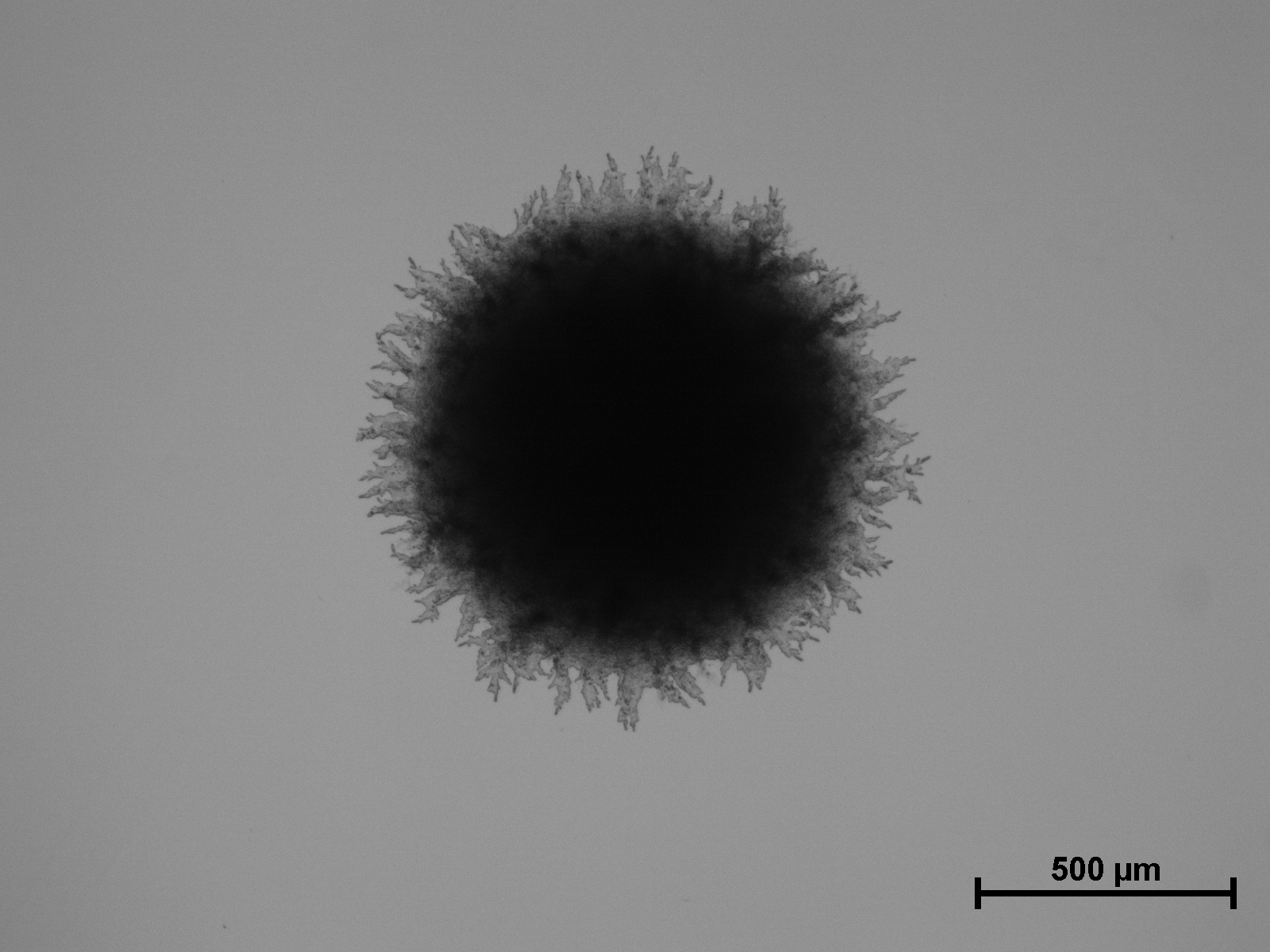

Supplement: S1 Data — Sample images from a single colony for processing by TAMMiCol. (ZIP) [file pcbi.1006629.s003.zip › Test Data/AWRI 796 PLOS 50uM s5 4X 115h.tif]

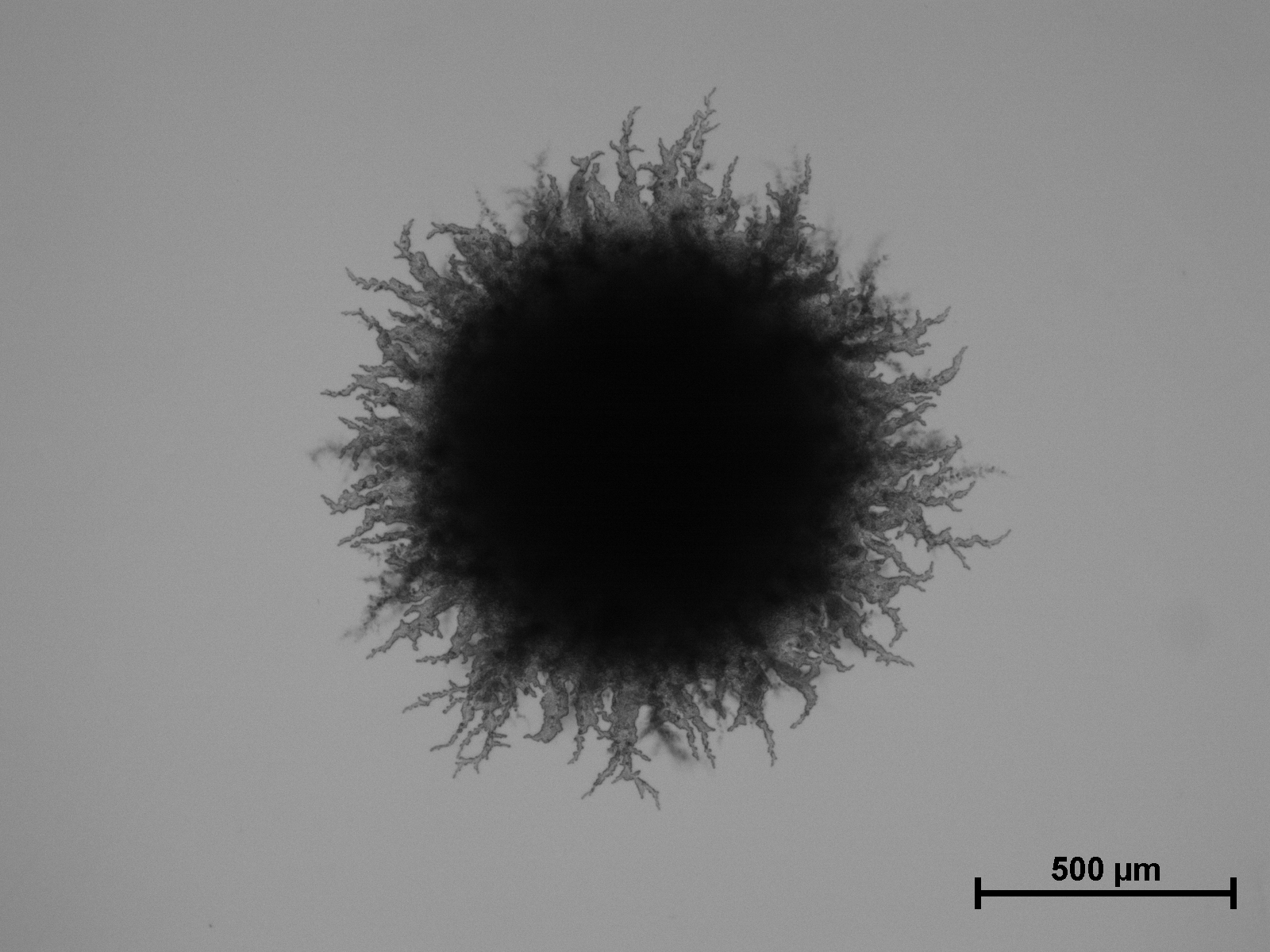

Supplement: S1 Data — Sample images from a single colony for processing by TAMMiCol. (ZIP) [file pcbi.1006629.s003.zip › Test Data/AWRI 796 PLOS 50uM s5 4X 162h.tif]

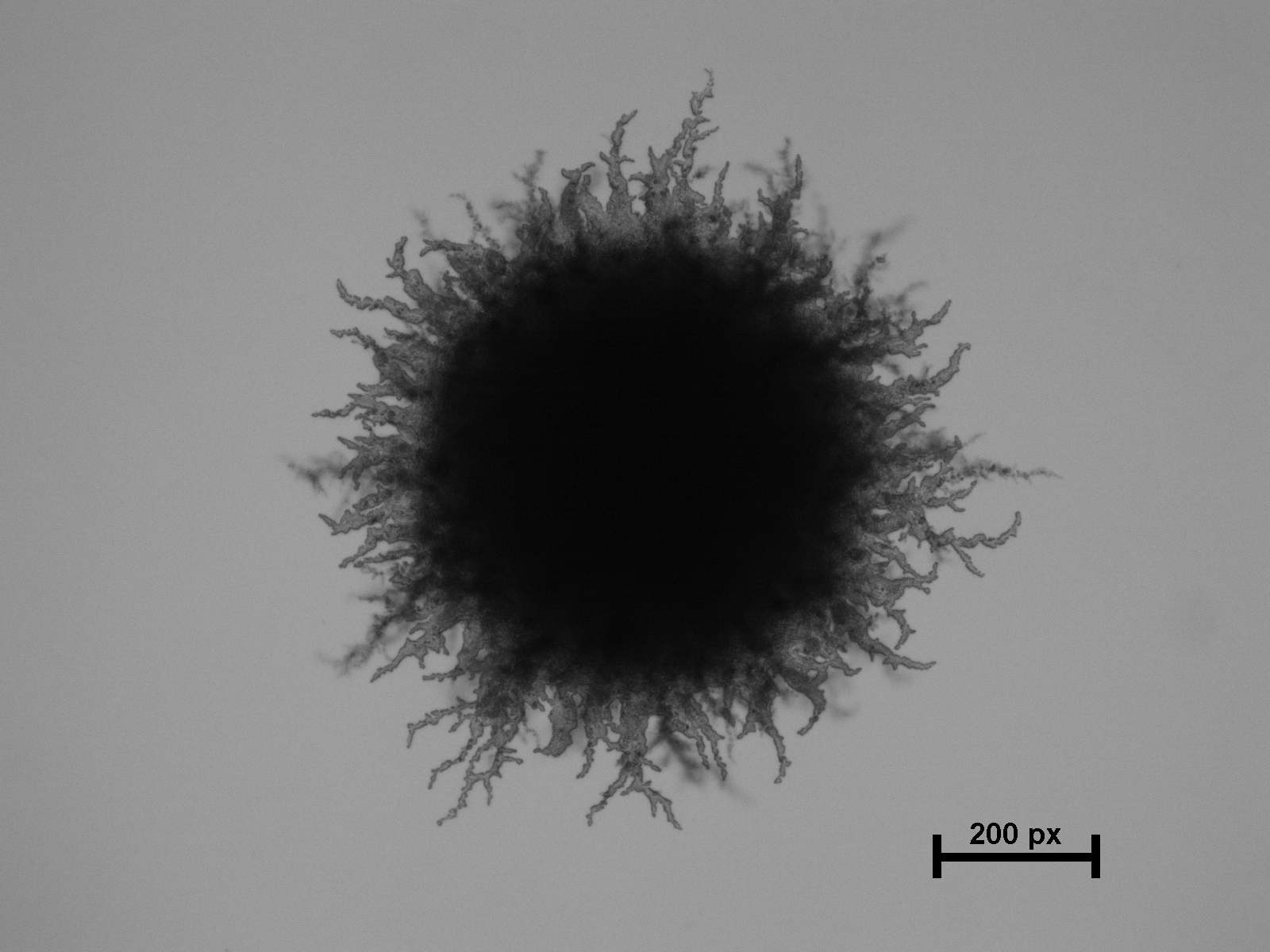

Supplement: S1 Data — Sample images from a single colony for processing by TAMMiCol. (ZIP) [file pcbi.1006629.s003.zip › Test Data/AWRI 796 PLOS 50uM s5 4X 211h.tif]

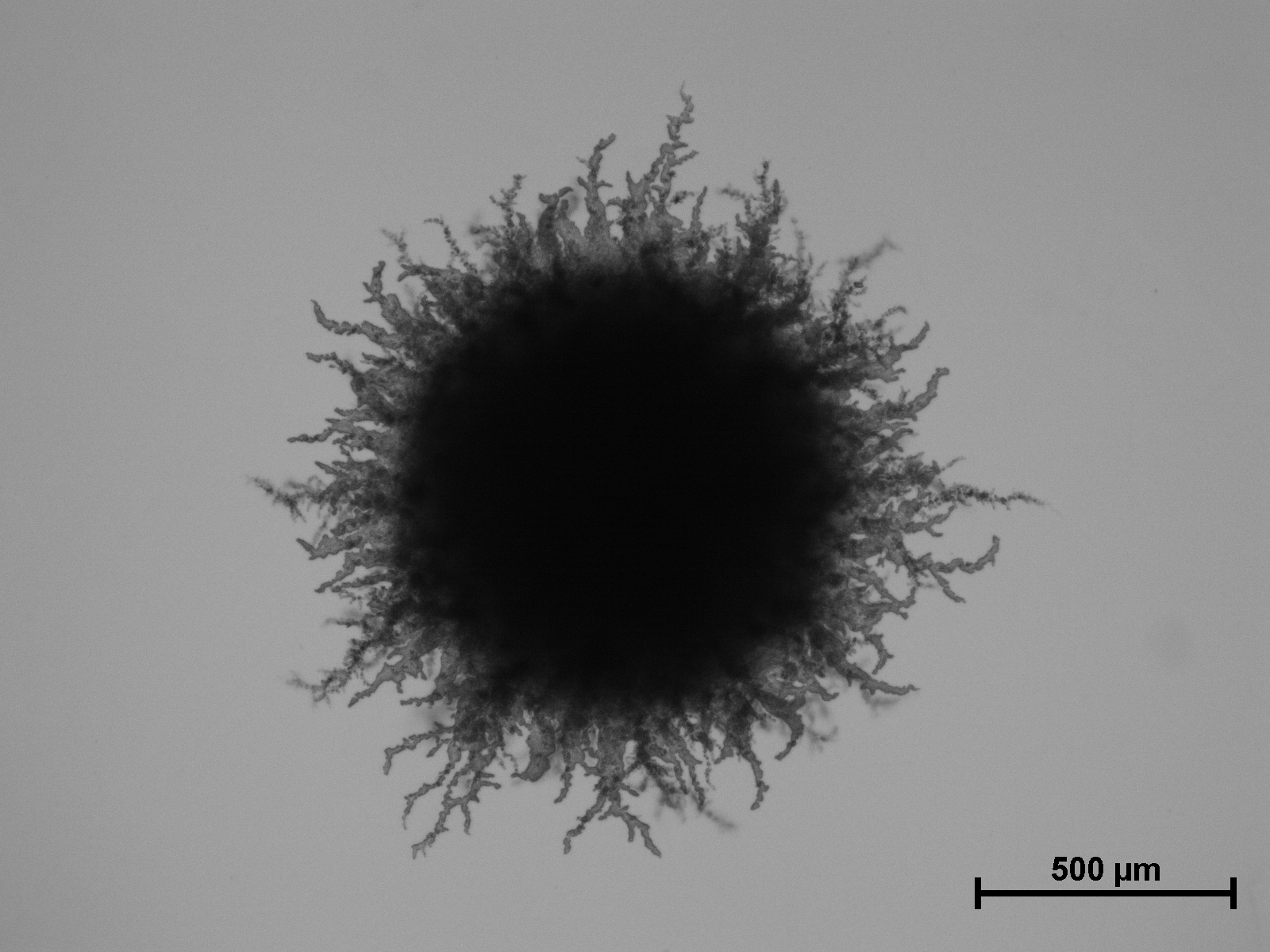

Supplement: S1 Data — Sample images from a single colony for processing by TAMMiCol. (ZIP) [file pcbi.1006629.s003.zip › Test Data/AWRI 796 PLOS 50uM s5 4X 233h.tif]

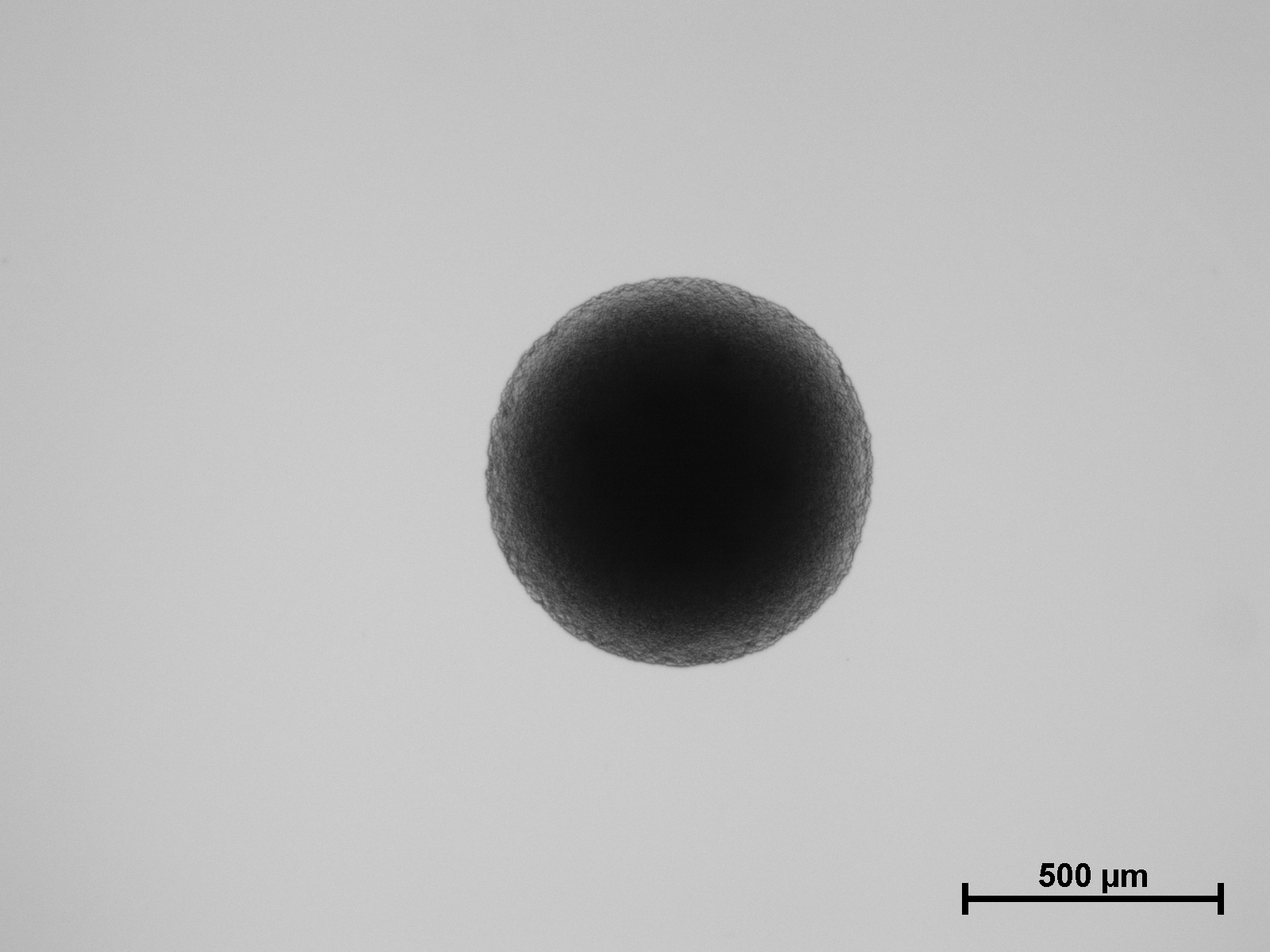

Supplement: S1 Data — Sample images from a single colony for processing by TAMMiCol. (ZIP) [file pcbi.1006629.s003.zip › Test Data/AWRI 796 PLOS 50uM s5 4X 73h.tif]

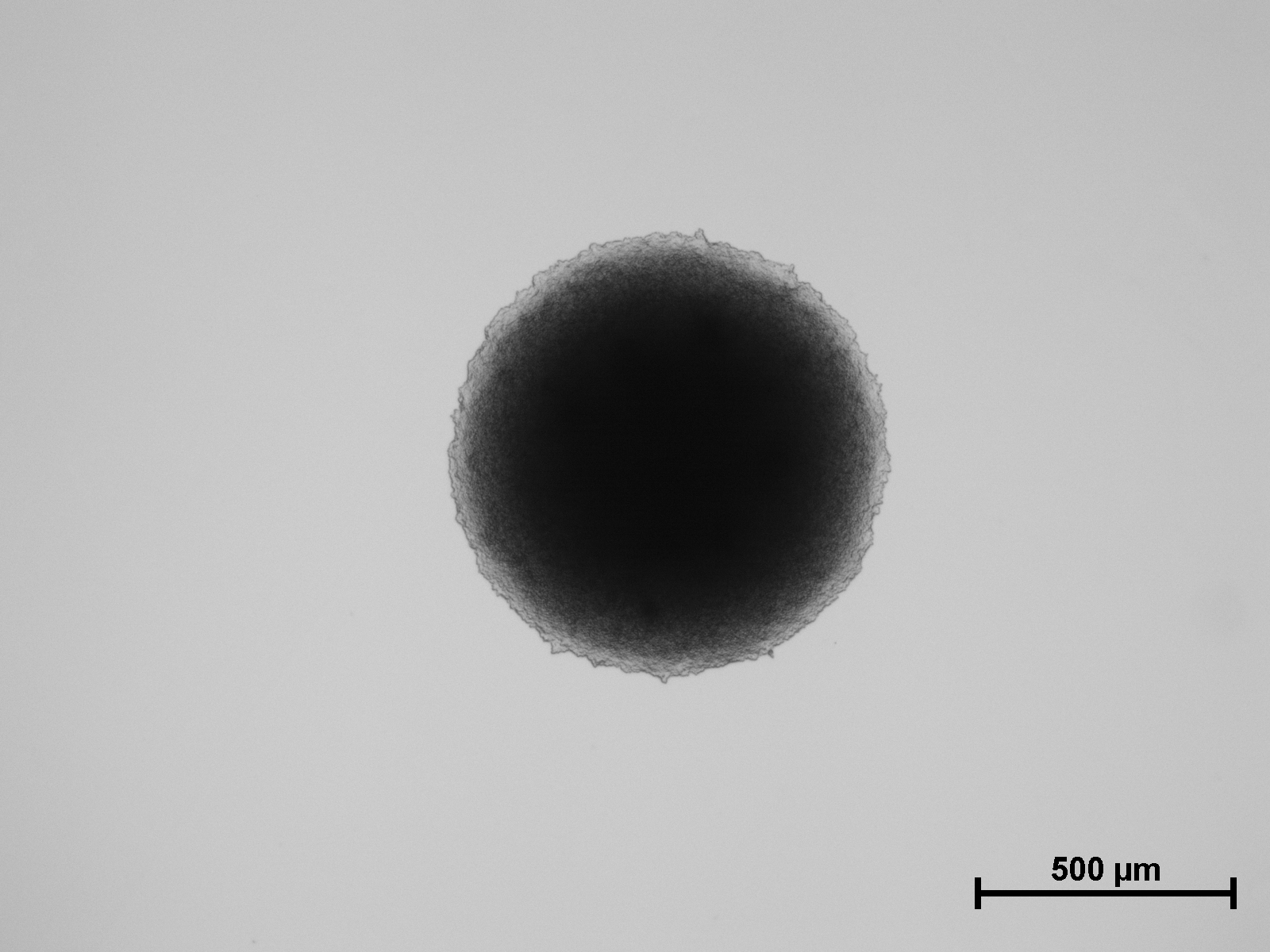

Supplement: S1 Data — Sample images from a single colony for processing by TAMMiCol. (ZIP) [file pcbi.1006629.s003.zip › Test Data/AWRI 796 PLOS 50uM s5 4X 87h.tif]
